# Inferring the demographic history of European *Ficedula* flycatcher populations

**DOI:** 10.1186/1471-2148-13-2

**Published:** 2013-01-02

**Authors:** Niclas Backström, Glenn-Peter Sætre, Hans Ellegren

**Affiliations:** 1 Department of Evolutionary Biology, Evolutionary Biology Centre (EBC), Uppsala University, Norbyvägen 18D, Uppsala, SE-752 36, Sweden; 2 Department of Organismic and Evolutionary Biology (OEB), Museum of Comparative Zoology (MCZ), Harvard University, 26 Oxford street, Cambridge, MA, 02138, USA; 3 Center for Ecological and Evolutionary Synthesis (CEES), Department of Biology, University of Oslo, P. O. Box 1066 Blindern, Oslo, N-0316, Norway

**Keywords:** *Ficedula* flycatchers, Demography, Differentiation, Gene-flow

## Abstract

**Background:**

Inference of population and species histories and population stratification using genetic data is important for discriminating between different speciation scenarios and for correct interpretation of genome scans for signs of adaptive evolution and trait association. Here we use data from 24 intronic loci re-sequenced in population samples of two closely related species, the pied flycatcher and the collared flycatcher.

**Results:**

We applied Isolation-Migration models, assignment analyses and estimated the genetic differentiation and diversity between species and between populations within species. The data indicate a divergence time between the species of <1 million years, significantly shorter than previous estimates using mtDNA, point to a scenario with unidirectional gene-flow from the pied flycatcher into the collared flycatcher and imply that barriers to hybridisation are still permeable in a recently established hybrid zone. Furthermore, we detect significant population stratification, predominantly between the Spanish population and other pied flycatcher populations.

**Conclusions:**

Our results provide further evidence for a divergence process where different genomic regions may be at different stages of speciation. We also conclude that forthcoming analyses of genotype-phenotype relations in these ecological model species should be designed to take population stratification into account.

## Background

Using genetic data to infer the demographic history of a species or a population is of importance for several reasons. For instance, a central goal in evolutionary genetics is to understand which forces have contributed to the observed patterns of genetic variation in natural populations. Of particular interest is the identification of genomic regions that evolve under pressure of natural selection and characterization of functional elements underlying fitness traits [1]. Since demographic events in the history of populations govern the distribution of alleles on a genome-wide scale, the design, analytical efficiency and interpretation of downstream population genetic analyses or genome scans to discover such regions can be enhanced if the population history is known in some detail [2-4]. For example, association analyses may be severely biased if there is population structure or recent admixture in the set of sampled individuals [5]. Moreover, demographic inference based on genetic data supplements morphological records in the quest towards understanding the natural history of organisms on deeper time scales [eg. [6] and can aid in discriminating between different scenarios of population differentiation and speciation, for instance, between speciation models including or excluding post-divergence gene-flow [7,8]. Until recently, demographic history and the factors governing genetic diversity were generally studied using limited data sets. However, since the variance in genetic diversity at a single or a few loci is unlikely to reflect the overall genomic patterns, assessment of the proportional contribution of drift, selection and demography in shaping genetic variability and population differentiation should ideally be based on multi-locus datasets [7,9,10]. A recently developed and powerful way of disentangling between alternative demographic hypotheses is the application of isolation migration model theory via a maximum likelihood analysis framework [eg. [11] of coalescence based models of population history [12]. This type of analysis can be time consuming and computer intense and are still not suitable for data on a genome scale but can be useful for multi-locus re-sequencing data sets with a moderate number of loci.

**Figure 1:**
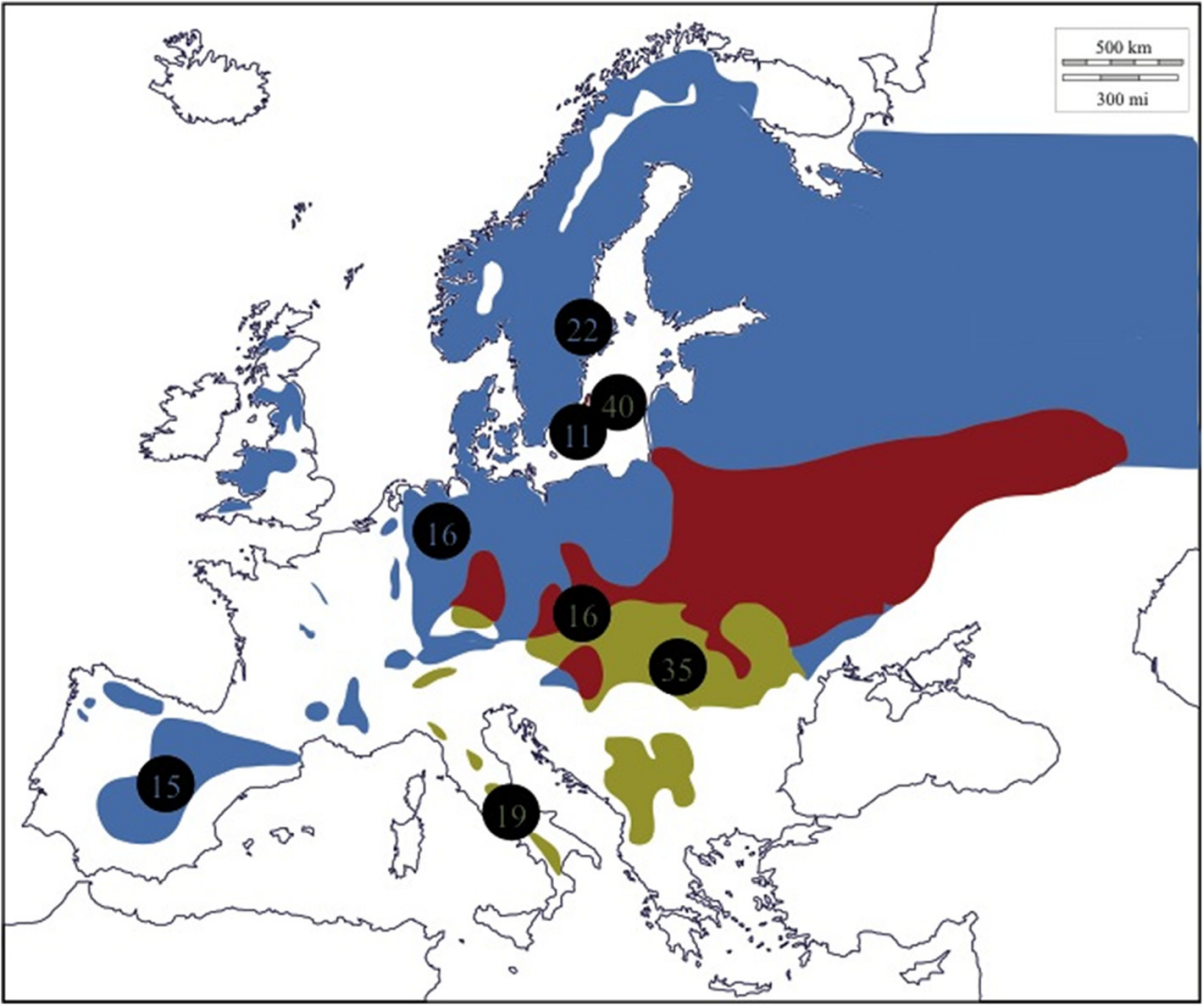
**European breeding distribution ranges for the pied flycatcher (*Ficedula hypoleuca***, **light blue)**, **the collared flycatcher (*Ficedula albicollis***, **green), and regions where both species occur together (red).** Unreservedly redrawn and adapted from range maps in Cramp & Perrins [27]. Circles indicate sampling sites and the number of birds collected on each site is given within the circle. Numbers for pied flycatchers are in blue font and numbers for collared flycatchers are in green font.

The pied flycatcher (*Ficedula hypoleuca*) and the collared flycatcher (*F. albicollis*) are small, migratory, passerine birds (family *Muscicapidae*) which occur over large parts of the western Palearctic (Figure 1). Current distribution ranges likely reflect expansions from Pleistocene glacial refuges on the Iberian (pied flycatcher) and the Apennine (collared flycatcher) peninsulas [13]. Areas of sympatric occurrence are present both in central and eastern Europe and on the Baltic Sea islands Gotland and Öland (Figure 1), and hybridization occurs at a low rate within these zones [14-16]. The hybrid zone on the islands in the Baltic Sea might have been formed as recently as 150 years (Gotland) to 50 years (Öland) ago when the collared flycatcher started colonizing the islands previously occupied by the pied flycatcher only [17,18]. The species system has been subject to thorough studies of speciation and hybridization, and the emerging consents include the presence of powerful intrinsic post-zygotic isolation (female hybrids are thought to be completely sterile), and potential reinforcement of pre-copulatory isolation, despite limited ecological differentiation [19,20]. Moreover, a suite of genetic mapping studies [21-25], transcriptome characterization [e.g. [26] and on-going efforts to sequence the flycatcher genome point towards that the species system is underway of becoming a genetic/genomic model and hold promise for downstream unravelling of important genotype-phenotype relationships. However, the knowledge about the demographic history is still sparse and likely insufficient to allow for robust interpretations of results from genome-wide selection scans or association efforts in these species.

We set out to use a multi-locus re-sequencing approach to obtain better understanding of the population history of the collared flycatcher and the pied flycatcher in Europe. Initially we focused on between-species divergence by estimating effective population sizes, species divergence time and by assessing potential post-divergence gene-flow. We followed up by investigating intra-specific patterns of genetic diversity and differentiation, our specific aims being to i) assess the level of genetic diversity in different parts of the distribution range of each species, ii) identify potential population structure within species, and iii) use allele frequency data to look for signs of demographic events in the history of the species.

## Methods

### Sampling and DNA extraction

Blood samples were available from previous studies [13,28,29] and taken during breeding season from between 11 and 40 birds at four different locations within each species distribution range, respectively (Figure 1). Pied flycatchers were sampled in central Spain (Madrid, 40° 24’ 0” N / 3° 41’ 0” W, n=15), northern Germany (Lingen, 52° 31’ 0” N / 7° 19’ 0” E, n=16), central Sweden (Uppsala, 59° 51’ 0” N / 17° 38’ 0” E, n=22) and in southeastern Sweden (Öland, 56° 40’ 0” N / 16° 22’ 0” E, n=11). Collared flycatchers were sampled in central Italy (Abruzzo, 42° 28’ 0” N / 14° 13’ 0” E, n=19), northern Hungary (Budapest, 47° 30’ 0” N / 19° 5’ 0’ E, n=35), sothern Czech Republic (Břeclav, 48° 46’ 0’ N / 16° 53’ 0” E, n=16) and in southeastern Sweden (Öland, 52° 31’ 0” N / 7° 19’ 0” E, n=40). DNA was extracted with either the DNEasy DNA extraction kit (QIAGEN), or with a standard proteinase K digestion, phenol-chloroform purification procedure [30].

### Marker development

We selected 24 autosomal, gene-based (intronic) markers from a set previously developed for amplification in a wide range of avian taxa [22]. The markers were chosen on basis of their amplification success in *Ficedula* flycatchers and to represent genes widely distributed over the genome, including both macro- and microchromosomes (Table 1). PCR amplifications were set up according to Backström *et al.*[22], the general recipe was a 20 μl reaction with 50–100 ng of template DNA with 50 μM of each dNTP, 2.5 mM MgCl_2_, 0.5 pmol of each primer, and 0.025 U of Ampli*Taq* or Ampli*Taq* Gold (Applied Biosciences). All PCRs were run on a Tetrad PTC-100 thermal cycler (MJ Research) with the general temperature profile; initial denaturation 5 min at 95°C, 20 cycles with 30 s denaturation at 95°C, 30 s annealing starting off at 65°C and decreasing the annealing temperature with 0.5°C per cycle and 1 min elongation at 72°C, 20 cycles with the same temperature profile but with fixed annealing temperature at 55°C, and finally finishing off with an extra elongation step for 5 minutes at 72°C. PCR products were purified with Exo-SAP IT (USB Corp.) according to recommendations from the manufacturer. Purified PCR products were prepared for sequencing by running a 30 cycle sequencing reaction using ≈100 ng product together with 3 pmol of either forward or reverse primer, 0.875 μl BDX64, 0.125 μl BigDye3.1, 1.5 μl 5X dilution buffer and 10 μl ddH_2_O (Applied Biosystems). The temperature profile was initiated by a denaturation step for 3 min at 96°C followed by 30 cycles including 10 s at 96°C, 5 s at 50°C and 2 min at 60°C as suggested by the manufacturer. Sequencing reactions were purified using the XTerminator system according to manufacturer’s protocol and sequences were run on an ABI3730xl DNA Analyzer (Applied Biosystems). Likely as a consequence of length polymorphisms in some loci in some populations, all loci could not be sequenced to full length in all individuals. The number of chromosomes sequenced for a specific population and a specific locus is given in Table 1.

**Table 1:** List of genes included in the study and the number of sequenced chromosomes per population and total for the species (Tot) for each locus

			**Pied flycatcher**					**Collared flycatcher**				
**Locus**	**ID**	**Length**	**Sp**	**Ge**	**Sc**	**Ba**	**Tot**	**It**	**Hu**	**Cz**	**Ba**	**Tot**
*PSMC2*	12884	967	18	22	16	20	76	34	60	24	60	178
*ARP6*	18851	611	26	32	30	22	110	38	64	22	74	198
*RABL4*	20454	309	28	28	28	20	104	38	66	30	6	140
*ETNK1*	21571	643	12	32	18	18	80	28	40	26	20	114
*ABHD10*	24813	1,008	16	30	22	20	88	28	30	28	70	156
*MOSPD2*	26743	757	2	22	0	20	44	6	40	8	74	128
*KIAA1706*	19789	105	4	22	18	22	66	6	38	6	78	128
*20904*	20904	487	16	30	22	22	90	34	36	24	46	140
*UNKNOWN*	13093	393	4	0	18	22	44	8	36	0	76	120
*CRIPT*	16264	666	4	30	18	22	74	12	38	20	74	144
*PSMB1*	18217	449	28	30	30	22	110	30	40	26	76	172
*PPIL4*	20195	431	16	24	20	18	78	30	34	22	62	148
*DST*	26267	829	6	14	0	0	20	20	4	20	72	116
*CBPZ*	25149	778	4	10	12	12	38	6	38	10	28	82
*08235*	08235	431	24	26	12	16	78	32	42	28	62	164
*PSMD14*	18142	325	4	0	12	20	36	6	36	0	80	122
*ADAL*	06419	509	4	0	20	20	44	6	32	0	20	58
*POLR2C*	01768	317	4	0	20	22	46	8	32	0	54	94
*UNKNOWN*	01304	458	20	32	16	20	88	26	36	26	68	156
*PSMD6*	11836	514	18	32	22	22	94	26	38	22	78	164
*HARS*	01152	703	22	20	22	22	86	36	68	30	74	208
*WDR24*	03862	759	20	14	22	16	72	30	58	30	72	190
*02419*	02419	532	4	0	16	22	42	8	32	0	22	62
*HEPACAM*	00574	183	22	22	30	18	92	36	40	26	58	160
**Sum**		**13,164**	**326**	**472**	**444**	**458**	**1,700**	**532**	**978**	**428**	**1,404**	**3,342**

Sp = Spain, Ge = Germany, Sc = Scandinavian mainland, Ba = Baltic Sea islands, It = Italy, Cz = Czech Republic. The ID given is the five last figures in the orthologous transcript identifier in the chicken genome assembly (ENSGALG000000xxxxx) according to Backström *et al.*[23].

### Data analysis

Sequences were edited using Sequencher 4.7 (Gene Codes Corp.) and trimmed to include only non-coding parts (introns). Locus specific alignments were created using Clustal W [31] as implemented in MEGA 5.0 [32]. We selected 10 individuals (20 chromosomes) with the highest sequence coverage as averaged over all 24 loci from each of the four populations from each species, respectively (40 birds in total per species), for analysis.

We applied a six-parameter isolation-migration model [IMa; [33] to the data using selections of individuals that represented two allopatric (separate analyses including Spanish pied flycatchers compared to Italian collared flycatchers and Hungarian collared flycatchers, respectively) and one sympatric (Baltic Sea islands pied flycatchers and Baltic Sea islands collared flycatchers) population pair as well as to a dataset including all 10 individuals from all four populations lumped together for each species, respectively. Since the isolation-migration model assumes no intra-locus recombination we inferred gametic phase and analysed all four datasets for signs of recombination using PHASE and the four-gamete test as implemented in DnaSP [34], and cut the alignments so that the longest stretch of sequence without evidence for recombination was kept for analysis. IMa simulates genealogies under different demographic scenarios using a Markov chain Monte Carlo approach and provides the estimates of six parameter values (q1 = population mutation rate for population 1, q2 = population mutation rate for population 2, qA = population mutation rate for the ancestral population, τ = time since divergence scaled by the mutation rate, m1 = migration rate from population 2 to population 1 scaled by the mutation rate and m2 = migration rate from population 1 to population 2 scaled by the mutation rate) that best fit the data. All datasets were analysed in an initial M-mode run sampling every 100 genealogies to a sum of 5*10^5^ genealogies after a burn-in of 1 million steps with prior ranges as follows: q1 = 0–25 q2 = 0–25, qA = 0–25, τ = 0–25, m1 = 0–15, m2 = 0–15. After inspecting the posterior distributions for the parameters a second, equally long, M-mode run was performed with narrower prior intervals and a different random seed number. The prior ranges for the second run were generally q1 = 0–1 q2 = 0–1, qA = 0–1, τ = 0–5, m1 = 0–10, m2 = 0–10. ESS values varied substantially between runs, the second analysis of allopatric populations (Spanish pied flycatchers and Hungarian collared flycatchers) had lower ESS values than the other analyses, but there was good agreement in HiPt values and posterior distribution ranges between independent runs. Subsequent analysis and the interpretations were therefore restricted to the runs with narrower priors only. All parameter estimates were scaled by a mutation rate of 1.4*10^-9^[35] and a generation time of one year. We evaluated different demographic models by comparing relevant nested models (L-mode) to the full six-parameter model and assessed the significance by likelihood ratio tests implemented in the software [33].

Population genetic analyses including estimates of nucleotide diversity (π), Tajima’s *D*, and inter-population genetic differentiation (*F*_*ST*_) within and between species were calculated in DnaSP version 5 [34]. All single nucleotide polymorphisms (SNPs, 272 in collared flycatcher and 193 in pied flycatcher) were used to assess intra-specific population structure using both a model-based clustering algorithm based on allele sharing among populations [STRUCTURE v2.3; [36,37], and a principal component analysis (PCA) based population stratification tool in the software package eigensoft [SMARTPCA; [38]. STRUCTURE version 2.3 [36] was run with default settings, using the admixture model and inferring α, for 400,000 steps after a burn-in period of 100,000 steps for each species, respectively. For both species, 10 independent analyses with different random seeds were run for each value of K from K = 1 to K = 4. The optimal number of populations (K) was assessed using the method suggested in the STRUCTURE 2.3 documentation (http://pritch.bsd.uchicago.edu/software/documentation.pdf). Graphical displays of individual assignment coefficients were created using Distruct version 1.1 [39]. Nexus files exported from MEGA 5.0 [32] including intra-specific polymorphisms (see numbers above) were transformed to eigenstrat format using an in-house developed python script (Nexus2smartpca_v2.py, Charles Chapus personal communication). SMARTPCA [38] was run with default settings for each of the species separately as well as for both species combined, and the three most informative principal components were selected for the plots.

## Results

We collected up to 13,164 bp of sequence data from 24 autosomal loci (Table 1) for a total of 64 pied flycatchers and 110 collared flycatchers sampled from four different locations throughout their respective breeding ranges (Figure 1, Table 1). From each population, 10 individuals with the highest yield of sequence data were selected for analysis.

### Species divergence

**Figure 2:**
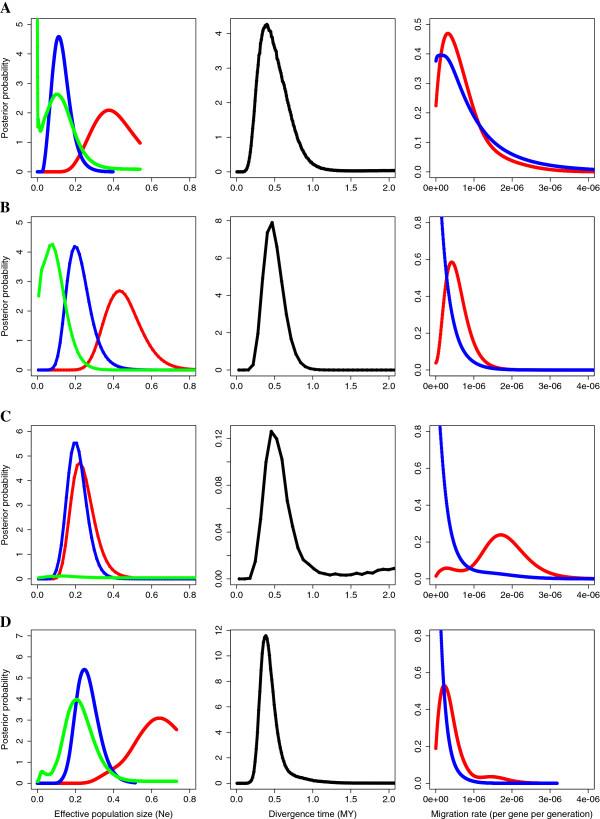
**Posterior probability distributions of the six parameters estimated using the isolation-migration model.** Left panel is current (pied flycatcher = blue, collared flycatcher = red) and ancestral (green) effective population size estimates in millions, middle panel is time of divergence in million years and right panel is post-divergence migration rates (per gene per generation) from pied flycatcher to collared flycatcher (red) and from collared flycatcher to pied flycatcher (blue). **A)** allopatric Spanish pied flycatcher and Italian collared flycatcher, **B)** allopatric Spanish pied flycatcher and Hungarian collared flycatcher, **C)** sympatric pied flycatcher and collared flycatcher from the Baltic Sea islands and **D)** between species comparison including data from all populations within each species.

The isolation-migration analyses showed relatively consistent results over different datasets including allopatric populations, sympatric populations and all populations from each species combined. Distributions of divergence time estimates from all pair-wise comparisons steadily fell in the range of a few hundred thousand to one million years and all analyses showed a biased migration rate with higher estimated gene-flow from the pied flycatcher into the collared flycatcher (eg. 0.22*10^-6^ gene^-1^ generation^-1^ for the analysis of all populations combined) than vice versa (0.0016*10^-6^ gene^-1^ generation^-1^). The rate of gene-flow from the pied flycatcher into the collared flycatcher was also considerably higher in the comparison of sympatric populations (1.70*10^-6^ gene^-1^ generation^-1^) than in the comparisons of allopatric populations (0.31 - 0.42*10^-6^ gene^-1^ generation^-1^). The analysis of samples from all populations showed that the estimated effective population size was higher for the collared flycatcher (N_e_ ≈ 450,000 – 750,000) than for the pied flycatcher (N_e_ ≈ 150,000 – 400,000) and for the ancestral population (N_e_ ≈ 20,000 – 400,000). Posterior probability distributions for all parameter estimates and datasets are presented in Figure 2 and HiPt (Maximum likelihood) and 90% highest posterior density boundaries in Table 2.

**Table 2:** HiPt (Maximum Likelihood) and HPD90 (90% Highest Posterior Density) estimates from the isolation-migration (IMa) analysis

**Population pair**	**Pied N**_**e**_	**Collared N**_**e**_	**Ancestral N**_**e**_	**Divergence time (years)**	**Pied ⇒ Collared (10**^**-6**^**)**	**Collared ⇒ Pied (10**^**-6**^**)**
**Spanish - Italian**						
HiPt	112,300	376,000	257	396,000	0.31	0.14
HPD90low	50,700	257,200	257	108,000	0.0024	0.0023
HPD90high	201,000	533,100	250,700	1,167,500	1.53	2.16
**Spanish - Hungarian**						
HiPt	195,500	431,100	80,400	454,800	0.42	0.0050
HPD90low	107,200	270,300	7,300	218,000	0.095	0.0050
HPD90high	359,500	650,200	197,300	918,300	1.03	0.66
**Baltic Sea islands**						
HiPt	205,500	225,000	131,800	454,800	1.70	0.0045
HPD90low	112,700	116,400	7,800	314,900	0.15	0.0045
HPD90high	338,200	395,600	13,907,400	69,940,800	2.82	1.15
**All populations**						
HiPt	246,800	640,200	205,800	384,700	0.22	0.0016
HPD90low	167,100	439,200	22,300	133,500	0.0016	0.0015
HPD90high	361,700	730,900	387,200	1,138,400	1.44	0.42

N_e_ = effective population size.

Direction of gene-flow is indicated by an arrow (⇒).

Since the analysis of allopatric Spanish pied flycatchers and Hungarian collared flycatchers showed low ESS values (Effective Sample Size, an indication about the number of independent estimates that have been generated for each parameter) we focused on the other three datasets for the interpretation of nested models. These analyses supported the findings from the isolation-migration analysis with only minor differences among data sets (Additional file 1 Supplementary Information, bracketed parameters are collared flycatcher N_e_, pied flycatcher N_e_, ancestral N_e_, migration rate, migration rate from pied to collared flycatcher and migration rate from collared to pied flycatcher, e.g. the full model being (ABCDE)). In general, a model assuming equal effective population sizes between the pied flycatcher and the collared flycatcher (AABXX) or between the collared flycatcher and the ancestral flycatcher (ABAXX) was significantly rejected, especially if the gene-flow was assumed to be equal in both directions (AABDD, ABADD) or completely absent (AAB00, ABA00). A model assuming equal effective population sizes between the pied flycatcher and the ancestral flycatcher (ABBXX) was also rejected if the rate of post-divergence gene-flow was forced to be equal in both directions (ABBDD), but not otherwise (ABBDE). The models excluding gene-flow in both directions (XXX00) were the least supported nested models of all, irrespective of if they allowed for differences in effective population size or not. Specifically, the full model, allowing for unequal population sizes and gene-flow in both directions (ABCDE) was not significantly better than a model with no gene-flow from the collared flycatcher to the pied flycatcher (ABCD0) and could be rejected in all datasets. However, the corresponding model without gene-flow from the pied flycatcher to the collared flycatcher (ABC0D) was marginally, but significantly, rejected in both sympatry (p-value = 0.04) and in the comparison of all populations combined (p-value = 0.02).

### Polymorphisms and allele frequency distributions

The overall genetic diversity (π) was lower in the pied flycatcher (mean 0.0043 ± S.D. 0.0024) than in the collared flycatcher (0.0051 ± 0.0028), but the difference was not significant (Wilcoxon’s test, W = 227.5, p-value = 0.22). There were only small differences in genetic diversity between populations within species, the range of diversity estimates was between 0.0044 - 0.0049 for all populations with the exception of the Spanish pied flycatcher population which had lower diversity (0.0036; Table 3). Tajima’s *D* was significantly higher (Wilcoxon’s test, W = 148, p-value = 0.006) in the pied flycatcher (0.18 ± 0.56) than in the collared flycatcher (−0.32 ± 0.69) but there was no significant difference between populations within species (Table 3).

**Table 3:** **Locus specific and average nucleotide diversity (π) and Tajima’s *D *estimates for each population separately and summarized over all populations within species (Tot)**

	**Nucleotide diversity (π, ‰)**										**Tajima’s *D***									
	**Pied flycatcher**					**Collared flycatcher**					**Pied flycatcher**					**Collared flycatcher**				
**Locus**	**Sp**	**Ge**	**Sc**	**Ba**	**Tot**	**It**	**Hu**	**Cz**	**Ba**	**Tot**	**Sp**	**Ge**	**Sc**	**Ba**	**Tot**	**It**	**Hu**	**Cz**	**Ba**	**Tot**
*PSMC2*	0.0	2.3	2.1	2.6	2.3	2.1	2.9	3.5	2.1	2.7		0.12	0.03	0.58	0.02	−0.49	−0.47	−0.29	−0.72	−1.05
*ARP6*	3.4	4.1	3.0	5.7	5.3	6.4	8.9	6.4	5.3	7.5	−0.21	0.27	−0.82	1.18	−0.01	0.58	0.55	0.52	1.26	0.91
*RABL4*	10.2	8.6	8.5	9.2	9.1	4.7	9.1	6.4	6.7	9.5	1.54	0.85	0.60	1.43	0.40	−0.09	0.39	−0.10	0.15	−0.12
*ETNK1*	4.7	2.4	2.1	2.1	2.6	2.9	4.1	4.7	2.8	3.8	−0.39	0.72	0.27	0.17	−0.59	−1.05	−0.16	0.41	−0.38	−0.92
*ABHD10*	5.8	4.1	5.2	5.6	5.8	6.0	5.5	4.9	4.5	5.3	0.11	0.17	0.37	−0.13	0.27	0.47	0.81	−0.37	−0.31	−0.14
*MOSPD2*	4.1	3.6		4.4	5.0	6.4	5.9	5.6	5.2	7.2	0.00	0.33		0.64	0.44	1.39	0.56	1.10	−0.31	0.60
*KIAA1706*	0.0	3.5	3.5	3.0	3.7	5.1	4.7	0.0	4.8	5.4		0.59	0.49	0.24	1.06	0.85	0.98		−0.34	−0.28
*20904*	4.6	5.9	5.3	5.2	5.7	5.1	4.5	5.5	6.1	5.5	0.74	0.78	0.38	2.53	0.57	0.31	−0.32	−1.03	−0.42	−0.75
*UNKNOWN*	4.4		3.9	5.1	4.8	2.9	3.8	0.0	3.7	4.1	0.17		0.83	1.31	0.83	1.62	0.39		0.11	−0.01
*CRIPT*	4.4	3.9	6.6	4.9	4.4	6.4	5.0	5.4	5.5	5.9	−0.80	1.00	0.64	0.50	0.31	0.22	−0.21	0.37	−0.07	−0.46
*PSMB1*	1.5	5.7	6.9	5.1	3.1	2.4	4.7	3.0	2.3	2.6	−0.99	−0.01	1.49	0.35	0.78	−0.16	0.27	0.68	−0.67	−0.43
*PPIL4*	5.7	6.1	2.9	3.9	5.3	3.4	2.5	3.4	2.9	4.8	1.84	0.17	0.19	−0.28	0.48	−0.66	−1.44	−0.83	−0.87	−0.24
*DST*	0.0	1.1			1.0	2.5	2.5	4.1	4.4	3.7		0.04			−0.29	−0.74	1.89	0.12	0.52	−0.20
*CBPZ*	0.8	5.1	4.3	3.5	3.5	4.4	5.9	6.8	4.8	6.6	−0.61	0.43	0.38	−0.88	−0.09	0.38	−0.39	0.39	−0.44	0.17
*08235*	5.5	2.9	4.5	5.6	4.8	6.9	7.0	5.3	5.2	5.6	−0.42	−0.30	0.55	−1.13	−1.04	−0.19	−0.80	−0.69	−0.18	−0.69
*PSMD14*	7.2		7.1	8.6	8.0	9.4	4.5		4.5	5.1	−1.38		0.64	0.19	0.46	−0.50	−0.99		−0.75	−0.83
*ADAL*	1.3		5.8	4.4	5.2	7.5	5.4		5	5.6	−0.61		1.07	1.02	1.15	0.01	0.66		0.83	0.42
*POLR2C*	0.0		2.3	2.7	2.3	0.8	0.0		1.2	0.8			−1.03	−0.66	−0.51	−1.05			−0.96	−1.42
*UNKNOWN*	1.8	2.6	2.2	1.4	2.2	5.8	4.5	4.7	5	5.5	0.87	−0.37	−0.75	−0.66	−0.51	−0.01	−0.77	−0.52	−0.28	−0.83
*PSMD6*	2.1	2.2	2.3	2.1	2.4	2.5	3.0	2.1	3.6	3.1	0.49	1.10	1.11	0.75	0.32	−0.92	−0.22	−1.02	−0.39	−0.89
*HARS*	0.0	0.3	0.0	0.0	0.0	0.0	0.1	0.0	1.3	0.6		−1.16					−1.07		−1.13	−1.30
*WDR24*	7.7	6.1	5.5	4.2	6.1	7.6	5.7	4.1	5.6	6.5	0.14	0.13	−0.43	0.23	−0.28	−0.26	−0.57	−0.44	−0.34	−0.42
*02419*	1.5		0.9	1.5	1.4	2.4	1.7		1.6	1.7	1.63		1.03	−0.81	−0.18	0.20	−0.34		−1.15	−0.38
*HEPACAM*	9.0	3.2	10.9	4.8	9.2	13.6	13	13.2	11.8	13.5	−0.01	−0.75	0.91	1.21	0.53	2.76	1.29	1.58	1.63	1.53
**Average**	**3.6**	**3.9**	**4.4**	**4.1**	**4.3**	**4.9**	**4.8**	**4.7**	**4.4**	**5.1**	**0.11**	**0.22**	**0.38**	**0.35**	**0.12**	**0.12**	**0.00**	**−0.01**	**−0.22**	**−0.32**

Sp = Spain, Ge = Germany, Sc = Scandinavian mainland, Ba = Baltic Sea islands, It = Italy, Hu = Hungary, Cz = Czech Republic.

### Population differentiation

The average *F*_*ST*_ between species was 0.31 ± 0.22. Within the pied flycatcher, there was significant differentiation between population pairs including the Spanish population with *F*_*ST*_ = 0.13 (p-value < 0.01) in the comparison to the Baltic Sea islands and the Scandinavian mainland populations and *F*_*ST*_ = 0.09 (p-value < 0.01) in the comparison to the German population. The level of differentiation between all other population pairs was limited (*F*_*ST*_ < 0.01, p-value >0.05; Table 3). There was no significant differentiation between any collared flycatcher population pairs (*F*_*ST*_ in the range of 0 – 0.05, p-values > 0.05; Table 4).

**Table 4:** ***F***_***ST***_**– values (below diagonal) and standard deviation (above diagonal) for all population pairs as summarized over all 24 loci**

	**Pied flycatcher**				**Collared flycatcher**			
	**Sp**	**Ge**	**Sc**	**Ba**	**It**	**Hu**	**Cz**	**Ba**
**Sp**		**0.24**	**0.17**	**0.21**	0.25	0.22	0.23	0.23
**Ge**	**0.09**		**0.12**	**0.07**	0.22	0.18	0.21	0.16
**Sc**	**0.13**	**0.01**		**0.08**	0.23	0.19	0.25	0.21
**Ba (pied)**	**0.13**	**−0.01**	**0.01**		0.23	0.20	0.26	0.23
**It**	0.32	0.33	0.28	0.28		**0.07**	**0.13**	**0.09**
**Hu**	0.32	0.36	0.33	0.32	**0.02**		**0.08**	**0.12**
**Cz**	0.29	0.36	0.35	0.35	**0.05**	**0.01**		**0.11**
**Ba (coll.)**	0.26	0.29	0.27	0.25	**0.02**	**−0.03**	**−0.02**	

Sp = Spain, Ge = Germany, Sc = Scandinavian mainland, Ba = Baltic Sea islands, It = Italy, Hu = Hungary, Cz = Czech Republic. Intra-specific comparisons are in bold face font.

**Figure 3:**
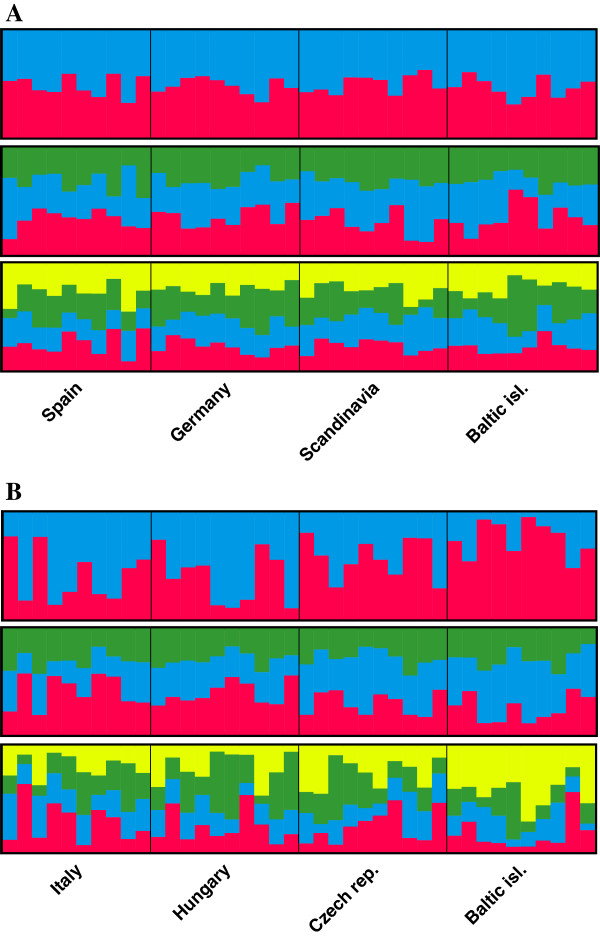
**The bars show assignments of individuals to populations as suggested by the STRUCTURE analysis for A) pied flycatcher populations and B) collared flycatcher populations.** The three panels for each species represent results from independent runs with K = 2 (top), K = 3 (middle) and K = 4 (bottom). One vertical bar represents one individual and the proportional assignment of each individual to a specific population is coded by color. The population origin of the samples is specified below bars.

**Figure 4:**
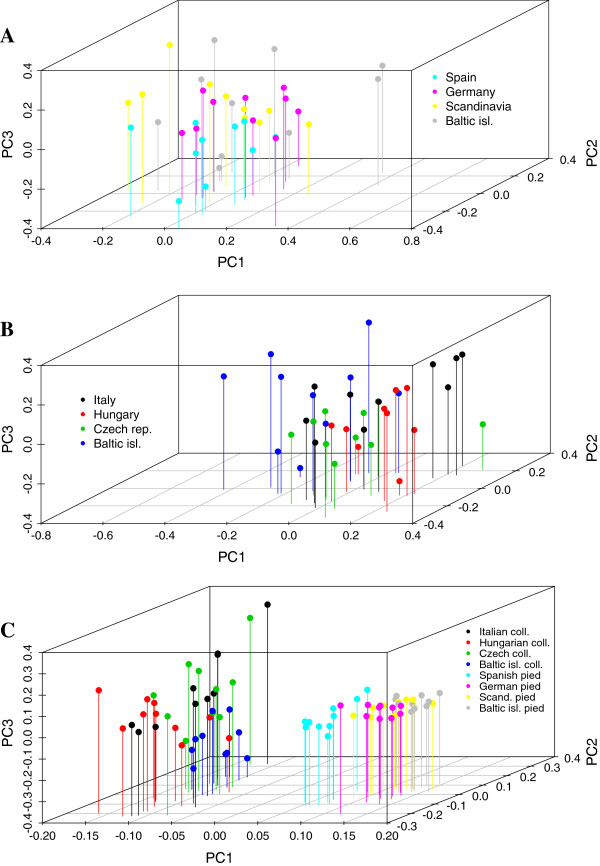
**Plot of the principal component analysis with SMARTPCA including the three most informative principal components for each dataset: A)data from pied flycatchers only,B)data from collared flycatchers only andC)data from both species combined.**

The analysis of assignment of individuals to specific populations with STRUCTURE [36] and SMARTPCA [38] did not indicate any considerable population stratification in either of the two species (Figure 3). Likelihood values for different number of clusters did not vary significantly and we interpreted the most likely number of clusters to be one in both the pied and the collared flycatcher. The PCA analysis also indicated limited population differentiation in both the pied flycatcher and the collared flycatcher. However, the resolution of the PCA allowed for more detailed visual inspection and a few observations are worth bringing up. First, in agreement with the intra-specific differentiation analyses using *F*_*ST*-_values, the Spanish pied flycatcher population grouped outside the range of the other pied flycatcher populations, in particular along the axis of principal component 2 and 3 (Figure 4A). Second, in the collared flycatcher the most differentiated population was the population from the Baltic Sea islands (Figure 4B). Both these patterns could also be observed when all individuals from both species were analyzed together (Figure 4C).

## Discussion

We re-sequenced 24 autosomal loci in population samples of pied flycatchers and collared flycatchers collected throughout their respective European breeding distribution ranges and used the data to infer parameters in the history of the species and to evaluate potential population stratification within species. The isolation-migration model fitting generated relatively consistent estimates for the parameter values over all data sets. The ancestral population size was generally estimated to be smaller than the current population sizes or similar to the current population size of the pied flycatcher. In agreement with the diversity estimates, the effective population size of the pied flycatcher was smaller than the effective population size of the collared flycatcher in all comparisons. Although the exact numbers for the effective population size estimates from the isolation-migration analysis should be treated with care since they are heavily dependent on the assumed generation time and mutation rate, it is of some interest to compare the ratio of census to effective population size estimates for the different species. The estimated census population size for the pied flycatcher, 5,250,000 in Europe plus >3,000,000 in Russia [40], is approximately one order of magnitude higher than the estimated effective population size. In the collared flycatcher, the estimated census population size is only about 10% of the pied flycatcher estimate 340,000-762,000, [40] and very similar to the estimated effective population size. In line with indications of a recent population expansion [17], this suggests a stronger bottleneck and a more dramatic population growth from the bottlenecked population in the pied flycatcher (ie. smaller refugial populations but significant recent population growth), during re-colonization of Northern Europe subsequent to the retrieval of the ice cover from the latest Pleistocene glaciations. Previous work has shown that the pied flycatcher is more opportunistic in choice of habitat than the collared flycatcher. For example, it can breed successfully also in relatively poor habitats [17,41], and pied flycatcher hatchlings are less vulnerable to periods of low food abundance than collared flycatcher hatchlings [42]. Hence, the pied flycatcher is likely able to re-colonize new areas more rapidly than the collared flycatcher when an ice cover retracts after a glaciation. This ability can potentially explain the current, more widespread and more northern distribution of the pied flycatchers as compared to the collared flycatcher.

There was also regularity in the estimated divergence time among datasets, all results pointed towards numbers around 0.5 million years. Previous estimates, using mitochondrial data and a fixed clock, implied a divergence time of 1–2 million years between the species (approximately 3% mtDNA divergence, [13]). The inconsistency between estimates could be caused by several factors [cf. [43]. First, the estimate from the isolation-migration model is dependent on the assumed generation time and mutation rates; a halving of the mutation rate would double the divergence time estimate. Second, divergence times based on strict molecular clocks have proven to be subject to biases from rate heterogeneity both among lineages and among genomic regions and the rate of mtDNA divergence is variable among different taxa [44]. Finally, the estimates could reflect a true difference in divergence between mtDNA and nuclear genes. During a speciation process different genomic regions may diverge at varying rates. Simulation analyses show that a higher degree of mtDNA differentiation is expected in organisms with female biased dispersal (eg. birds in general) due to smaller drift effects of introgressed alleles in regions with high intra-specific gene-flow [45]. The rate of differentiation in a region can also be dependent on the proximity (degree of genetic linkage) to selected alleles [46-48], differences in relative fitness of male and female hybrids [13], or the effective population size of the locus [49]. It is also known that female hybrids are sterile while male hybrids can produce viable offspring [16], albeit with severely reduced fitness [50]. This fitness difference has potentially contributed to reduce the gene-flow on mitochondria compared to autosomes [51]. Moreover, as mtDNA is clonally and strictly maternally inherited, recessive mutations are not masked by dominance, similar to the case for sex-chromosomes in hemizygous state [cf. Haldane’s rule; [52]. Consequently, there is scope for stronger selection against incompatible combinations of alleles and potential for more rapid build-up of barriers to gene-flow on mitochondria than on autosomes. If so, it is possible that recurrent secondary contact events following re-colonizations during warmer interglacial epochs have resulted in less introgression on the mitochondria than on the autosomes, similar to what has recently been observed on the Z-chromosome in several avian taxa [20,53-57]. This would result in relatively larger proportion of shared polymorphisms and, hence, a shallower divergence estimate for the autosomes. Another possible explanation for a deeper mtDNA divergence estimate is historical (but post initial divergence) introgression of mtDNA into any of our focal species from more distant relatives, ie. Atlas flycatcher (*Ficedula speculigera*) or semi-collared flycatcher (*F. semitorquata*) [51]. However, this scenario is less plausible since mtDNA from all abovementioned species are more or less equally differentiated postulating that past introgression has to have occurred from several extinct lineages into at least three of the extant species. Our data does not provide the power to disentangle the factors underlying the discrepancy between divergence time estimates based on mtDNA and genomic DNA. However, since many of the causes are not mutually exclusive, it is not unreasonable that a combination of factors have acted in concert, or during different time periods, to finally result in the observed pattern.

The strongest evidence for post-divergence gene-flow in our data was from the recently formed hybrid zone on the Baltic Sea islands. This result is in consistency with preceding studies [28,29] and further supports the idea that hybridization barriers are not yet complete in this young hybrid zone [20]. The data also support a directional bias with significantly more, or even completely unidirectional, gene-flow from the pied flycatcher to the collared flycatcher. This is in agreement with genotype information in backcrossing pied and collared flycatcher families [28] as well as re-sequencing data [29] and, given that hybrid females are sterile [20] this implies that the exclusive option for successful back-crossing will be hybrid males mating with collared flycatcher females.

Contrary to expectations from a substantial founder effect during re-colonization of new areas after expansion from glacial refuges, there was little variation in nucleotide diversity among populations within species. For the pied flycatcher the trend was actually opposite expectations, the Spanish population had the lowest diversity and the northern and thus more recently founded populations showed on average slightly higher diversity. This could reflect a smaller effective population size in Spain resulting from a partially fragmented population and, as implied by previous microsatellite data [58] and by our differentiation analyses, also probably from restricted gene-flow to other pied flycatcher populations. The Pyrenees may act as a dispersal barrier and the pied flycatcher has a disjunct distribution pattern in southwestern Europe indicating that there are limited opportunities for short-range diffusion. Overall, this suggests that newly established populations inhabiting novel habitats after glacial retraction were founded by a large number of immigrants. Alternatively, subsequent gene-flow between newly established and refugial populations may have been extensive enough to maintain similar diversity levels among populations.

All pied flycatcher populations showed slightly positive Tajima’s *D* values while the collared flycatcher populations showed on average slightly negative Tajima’s *D* values in derived populations but a slightly positive value in the supposedly ancestral Italian population. These observations stand in some contrast to previous findings in both the pied flycatcher and the collared flycatcher for a large set of Z-chromosome linked genes [average Tajima’s D = −0.52 and 0.14, respectively; 24] and to another dataset including both autosomal (*D* = −0.31 and 0.32) and Z-chromosome data D = 1.12 and −0.04; [28]. Admittedly, these mixed trends make interpretation difficult and speculative. It is known that historical events like bottlenecks followed by population expansions, can have varying effects on the allele frequency distribution dependent on the effective population size of the chromosomal region [59]. Since both species may have had fluctuating population sizes during relatively recent history, a discrepancy between autosomal and Z-chromosome linked loci is not entirely startling. However, the considerable variation among loci within chromosomes and the substantial difference between estimates among studies [24,28] suggest that the results spring from a complicated combination of random effects, demographic history and selective pressures unique to specific populations, species or genomic regions. This implies that particular caution should be taken before drawing any conclusions regarding general population history from these data.

The *F*_*ST*_ estimates varied only slightly among population comparisons between species and averaged at 0.31. This is lower than a previous estimate using a large set of Z-chromosome linked loci [0.36; [24], as expected given the lower effective population size [7,59], and reduced introgression on the Z-chromosome compared to the autosomes [53]. The latter effect could possibly be a consequence of the importance of Z-linked loci in species recognition and female mate choice [60]. Within species, the level of differentiation was generally low with the exception of pair-wise comparisons including the Spanish pied flycatcher population. This agrees with the analysis of genetic diversity and the principal component analyses, and implies that there is some differentiation among current European populations of the pied flycatcher that inhabit appreciably geographically separated areas.

Recent data from large-scale genotyping assays have revealed a potential to identify genetic structure on a very detailed scale [61]. For example, derived contemporary human populations, which actually harbor relatively limited genetic variability compared to eg. the species of flycatchers in our study [22,62], can be assigned to geographic sampling site with very high precision [61]. This indicates that the possibility to detect fine-scale population structure depends mainly on the number of markers used. It has also proven true that genome-wide scans for genotype-phenotype associations are highly sensitive to population structure [63], and that a large proportion of associations might be explained by rare genetic variants and alleles private to restricted populations [3]. Methods for handling stratification have been suggested and they apparently perform well except under some circumstances when the loss of power is substantial and when the number of markers is low [63]. Consequently, understanding the underlying population stratification is crucial to correctly infer the proportion and effect size of specific alleles on phenotypes of interest and to accurately transfer information from population to population. We utilized a limited number of markers, 24 loci with a few hundred SNPs in total, and detected significant population stratification in the pied flycatcher and observed some stratification also in the collared flycatcher, although between population differentiation, as measured by *F*_*ST*_, was insignificant in this species. Extensive genomic tools are currently under development in both the pied flycatcher and the collared flycatcher. Our data indicates that forthcoming analytical efforts using these tools should be designed so that within species stratification is taken into account using e.g. Genomic Control [63], or performed with individuals sampled within an unstructured subpopulation.

## Conclusions

We sequenced 24 autosomal intronic loci in population samples of the pied flycatcher and the collared flycatcher, two model species for ecology and behavior. The data indicate substantial differences in effective and census population size for the pied flycatcher, population stratification within species and shorter divergence time for autosomes than previously reported for mtDNA. We also observed unidirectional post-divergence gene-flow between the species. Our findings support a scenario where different portions of the genome can be at different stages of speciation and they provide important information about population stratification that will be useful for forthcoming analyses of the link between genotypes and phenotypes in these ecological model species.

### Availability of supporting data

All sequences have been deposited in the Dryad database under doi:http://dx.doi.org10.5061/dryad.dh53m.

## Competing interests

The authors declare no financial or non-financial competing interests.

## Authors’ contributions

NB and HE conceived of the study. NB designed the study, did the molecular work and analysed the data. GPS contributed materials. NB and HE wrote the manuscript with input from GPS. All authors approved the final version of the manuscript.

## Supplementary Material

Additional file 1 **Supplementary information: Nested model analysis test values (2LLR) and corresponding p-values.** df = degrees of freedom. * = 2LLR test statistic follows a mixed chi-squared distribution (Hey & Nielsen 2007). Allopatry 1 = Spanish pied flycatchers and Italian collared flycatchers, Allopatry 2 = Spanish pied flycatchers and Hungarian collared flycatchers, Sympatry = pied flycatchers and collared flycatchers from the Baltic Sea islands, Species = samples from all four populations combined for each species, respectively. (PDF 65 kb) Click here for file
