# Supplementary material for: Inferring the demographic history of European Ficedula flycatcher populations
Source: BMC Evol Biol. 2013 Jan 2;13:2. doi: 10.1186/1471-2148-13-2 (PMC3556140; doi:10.1186/1471-2148-13-2)
Supplement: Additional file 1 — Supplementary information: Nested model analysis test values (2LLR) and corresponding p-values. df = degrees of freedom. * = 2LLR test statistic follows a mixed chi-squared distribution (Hey & Nielsen 2007). Allopatry 1 = Spanish pied flycatchers and Italian collared flycatchers, Allopatry 2 = Spanish pied flycatchers and Hungarian collared flycatchers, Sympatry = pied flycatchers and collared flycatchers from the Baltic Sea islands, Species = samples from all four populations combined for each species, respectively. (PDF 65 kb) [file 1471-2148-13-2-S1.pdf]

Supplementary information

Nested model analysis test values (2LLR) and corresponding p-values. df = degrees of freedom. * = 2LLR test statistic follows a mixed chi-squared distribution (Hey & Nielsen 2007). Allopatry 1 = Spanish pied flycatchers and Italian collared flycatchers, Allopatry 2 = Spanish pied flycatchers and Hungarian collared flycatchers, Sympatry = pied flycatchers and collared flycatchers from the Baltic Sea islands, Species = samples from all four populations combined for each species, respectively.

		Allopatry 1		Allopatry 2		Sympatry		Species	
**Model**	**df**	**2LLR**	**pval**	**2LLR**	**pval**	**2LLR**	**pval**	**2LLR**	**pval**
ABCDD	1	4.4	0.04	168.6	0.00	5.9	0.02	7.1	0.01
ABCD0	1*	0.0	1.00	0.0	1.00	4.7	0.06	0.0	1.00
ABC0D	1*	1.2	0.28	925.1	0.00	5.5	0.04	7.3	0.02
ABC00	2*	683.5	0.00	925.1	0.00	27.7	0.00	921.7	0.00
AACDE	1	3.0	0.23	13.7	0.00	2.4	0.13	4.2	0.04
AAADE	2	3.0	0.23	374.9	0.00	5.7	0.06	4.3	0.12
AACDD	2	13.6	0.01	250.4	0.00	6.8	0.03	8.9	0.01
AAC00	3*	917.6	0.00	925.1	0.00	28.8	0.00	921.7	0.00
AAADD	3	13.6	0.00	647.6	0.00	11.0	0.01	8.9	0.03
AAA00	4*	917.5	0.00	925.1	0.00	48.6	0.00	921.7	0.00
ABADE	1	0.0	1.00	207.0	0.00	5.7	0.02	0.0	0.95
ABADD	2	4.7	0.10	459.5	0.00	9.9	0.01	8.2	0.02
ABA00	3*	683.4	0.00	925.1	0.00	47.6	0.00	921.7	0.00
ABBDE	1	0.0	1.00	365.2	0.00	5.7	0.02	0.1	0.77
ABBDD	2	4.7	0.09	641.4	0.00	8.2	0.02	7.2	0.03
ABB00	3*	683.3	0.00	925.1	0.00	44.4	0.00	921.7	0.00
